# COVID‐19 Infection Before or After Colorectal Cancer Diagnosis Is an Independent Predictor of Mortality and Treatment Delays Compared to Patients Who Never Tested Positive

**DOI:** 10.1002/cam4.71411

**Published:** 2025-11-25

**Authors:** Imran Qureshi, Steven Rella, Aasma Shaukat

**Affiliations:** ^1^ Department of Internal Medicine Rutgers New Jersey Medical School Newark New Jersey USA; ^2^ Division of Gastroenterology, Department of Medicine NYU Grossman School of Medicine New York New York USA

**Keywords:** colorectal cancer, COVID‐19, healthcare disparities, vaccination

## Abstract

**Background:**

Given COVID‐19's emergence as a new entity and colorectal cancer's (CRC) rising incidence in certain populations, we conducted this retrospective cohort study to determine the link between COVID‐19 and the mortality of those with CRC and how socioeconomic factors influence it.

**Methods:**

Using the National Cancer Database (NCDB), we used logistic regression to get the odds ratio (OR) for delayed treatment and Cox proportional hazards modeling for each stage to get the adjusted hazard ratios (HR) of mortality.

**Results:**

COVID‐19 positivity was associated with higher mortality and delayed treatment. The association of race, ethnicity, insurance, urbanization, comorbidity burden, education levels, and income varied by when the patient tested positive relative to colorectal cancer diagnosis.

**Conclusions:**

This implies that vaccinations may be a part of management and that CRC patients who develop COVID‐19 infection may warrant closer follow‐up during treatment.

COVID‐19 has dramatically transformed the landscape of medicine. According to the World Health Organization (WHO), COVID‐19 was responsible for over 1.8 million deaths worldwide in 2020, a number that newer estimates reveal may be even higher [1]. Although the acute phase of the pandemic has passed, COVID‐19 remains a significant disease that continues to impact individuals globally. Increasing evidence of its long‐term effects, commonly referred to as “long COVID,” suggests that people suffer from health issues even after recovering from the initial infection [2]. Both acute and post‐acute phases of COVID‐19 are linked to complications. Despite vaccination efforts and infection control protocols, all‐cause mortality due to COVID‐19 remains high even after the pandemic [3]. Colorectal cancer (CRC) is the fourth most common cancer in the U.S. [4]. Existing literature reveals how the pandemic disrupted CRC screening, leading to missed or delayed diagnoses and treatment [5, 6]. However, there is a lack of research on how COVID‐19 infection affects the incidence and progression of CRC. There is growing evidence of the oncogenic properties of COVID‐19. Mechanisms, such as inflammation, immune dysregulation, and viral genome integration, are being investigated as potential pathways through which COVID‐19 could influence tumorigenesis [7]. There is also evidence to suggest that cancer care for racial and ethnic minorities was disproportionately affected during the pandemic [8]. We conducted this retrospective cohort study to evaluate the association of COVID‐19 and its temporality to CRC diagnosis on treatment delays, mortality, and disparities in individuals over 18 affected by CRC compared to those who never tested positive.

We queried the National Cancer Database (NCDB) from 2020 and 2021 for patients with CRC, as it contained data regarding COVID‐19 testing based on preadmission or hospital testing from the site that reported the case. NCDB annually reports approximately 70% of newly diagnosed cancer cases from Commission on Cancer–accredited facilities in the U.S. [9]. Patients aged less than 18, not tested for COVID‐19, or who had unknown COVID‐19 test results were excluded. We divided our data into three categories: patients who never tested positive for COVID‐19, patients who tested positive for COVID‐19 for the first time before diagnosis of CRC, and patients who tested positive for COVID‐19 for the first time after diagnosis of CRC. We assessed sociodemographic factors at the time of CRC diagnosis and reported their frequencies. We used univariate and multivariate logistic regression to get the unadjusted and adjusted OR, respectively, along with a 95% confidence interval (CI) and *p*‐value for delayed treatment initiation, defined as > 6 weeks from diagnosis to initiation of any treatment modality. We fit a Cox proportional hazards model for each stage to get the univariate and multivariate HR of all‐cause mortality, with a 95% CI and *p*‐value, using patients who never tested positive for COVID‐19 as a reference. For multivariate regression analysis, we used sex, race, ethnicity, insurance status, setting, Charlson‐Deyo Comorbidity score, adults without a high‐school diploma in the patient's ZIP code, and median household income as covariates. Additionally, we assessed the effects of these factors on overall mortality for each of the two groups with COVID‐19 positivity using univariate and multivariate Cox regression. Our model met the proportional hazard assumption, which was tested using the Schoenfeld Residuals Test. We assessed overall survival using the Kaplan–Meier methods and their comparisons using the log‐rank test. An alpha level of 0.05 was considered significant. Statistical analysis was done using StataNow/MP 18.5 for Mac (StataCorp LLC, Texas, USA).

There were a total of 112,731 patients in our dataset, of which 103,913 (92.18%) never tested positive for COVID‐19, 2668 (2.37%) tested positive for COVID‐19 before diagnosis of CRC, and 6150 (5.46%) tested positive for COVID‐19 after diagnosis of CRC (Table 1). The median ages in our cohorts were 66 years (Q1–Q3: 55–76 years), 65 years (Q1–Q3: 53–76 years), and 63 years (Q1–Q3: 52–74 years), respectively. Those with COVID‐19 before diagnosis of CRC were diagnosed a median of 80 days prior (Q1–Q3: 20–203 days), whereas those with COVID‐19 after diagnosis were diagnosed a median of 118 days after (Q1–Q3: 44–241 days). We observed that those with COVID‐19 before diagnosis did not have a significant delay in receiving treatment for their malignancy (OR = 0.94, 95% CI = 0.84–1.05, *p* = 0.266), whereas those with COVID‐19 after diagnosis did (OR = 1.52, 95% CI = 1.43–1.63, *p* < 0.001) compared to those that never tested positive for COVID‐19. Both patients with COVID‐19 before and after diagnosis demonstrated an increase in all‐cause mortality (HR = 1.10, 95% CI = 1.00–1.20, *p* = 0.046 and HR = 1.18, 95% CI = 1.12–1.25, *p* < 0.001, respectively) compared to those that never tested positive for COVID‐19 (Figure 1). However, when stratified by stage of CRC, those with COVID‐19 after diagnosis of stage 1 (HR = 1.26, 95% CI = 1.01–1.56, *p* = 0.039), stage 2 (HR = 1.40, 95% CI = 1.21–1.61, *p* < 0.001), and stage 3 CRC (HR = 1.16, 95% CI = 1.04–1.30, *p* < 0.001) had increased mortality. No significant differences in mortality were noted in stage 4 CRC, regardless of COVID‐19 infection or in patients with COVID‐19 before diagnosis by stage of CRC.

**TABLE 1:** Frequencies of patient characteristics, unadjusted and adjusted odds ratios of delayed time to treatment initiation, and unadjusted and adjusted hazard ratios of mortality across different stages of CRC, followed by socioeconomic predictors of overall mortality.

	Frequencies			Regression analysis			
	No positive COVID test (*n* = 103,913)	COVID before diagnosis (*n* = 2668)	COVID after diagnosis (*n* = 6150)	COVID before diagnosis (*n* = 2668)		COVID after diagnosis (*n* = 6150)	
				Univariate logistic regression	Multivariate logistic regression	Univariate logistic regression	Multivariate logistic regression
Treatment delay	20,621 (21.75%)	495 (20.98%)	1687 (29.51%)	OR = 0.96 (95% CI = 0.86–1.06), *p* = 0.375	OR = 0.94 (95% CI = 0.84–1.05), *p* = 0.266	OR = 1.51 (95% CI = 1.42–1.60), *p* < 0.001	OR = 1.52 (95% CI = 1.43–1.63), *p* < 0.001*
				**Univariate Cox regression**	**Multivariate Cox regression**	**Univariate Cox regression**	**Multivariate Cox regression**
*Overall mortality*	27,654 (26.61%)	730 (27.36%)	1894 (30.80%)	HR = 1.08 (95% CI = 1.00–1.18), *p* = 0.056	HR = 1.10 (95% CI = 1.00–1.20), *p* = 0.046*	HR = 1.12 (95% CI = 1.07–1.18), *p* < 0.001	HR = 1.18 (95% CI = 1.12–1.25), *p* < 0.001*
Stage 1	17,524 (16.86%)	438 (16.42%)	768 (12.49%)	HR = 0.90 (95% CI = 0.65–1.24), *p* = 0.520	HR = 0.87 (95% CI = 0.62–1.23), *p* = 0.431	HR = 1.37 (95% CI = 1.13–1.66), *p* = 0.001*	HR = 1.26 (95% CI = 1.01–1.56), *p* = 0.039*
Stage 2	22,725 (21.87%)	529 (19.83%)	1148 (18.67%)	HR = 0.96 (95% CI = 0.77–1.21), *p* = 0.756	HR = 1.00 (95% CI = 0.78–1.30), *p* = 0.989	HR = 1.26 (95% CI = 1.10–1.44), *p* < 0.001	HR = 1.40 (95% CI = 1.21–1.61), *p* < 0.001*
Stage 3	27,890 (26.84%)	673 (25.22%)	1938 (31.51%)	HR = 1.09 (95% CI = 0.91–1.30), *p* = 0.336	HR = 1.10 (95% CI = 0.91–1.34), *p* = 0.317	HR = 1.08 (95% CI = 0.98–1.19), *p* = 0.138	HR = 1.16 (95% CI = 1.04–1.30), *p* < 0.001*
Stage 4	22,592 (21.74%)	622 (23.31%)	1600 (26.02%)	HR = 1.07 (95% CI = 0.96–1.19), *p* = 0.194	HR = 1.07 (95% CI = 0.96–1.21), *p* = 0.225	HR = 0.87 (95% CI = 0.81–0.93), *p* < 0.001	HR = 0.94 (95% CI = 0.87–1.01), *p* = 0.088
*Sex*							
Male	54,535 (52.48%)	1418 (53.15%)	3313 (53.87%)	(reference)	(reference)	(reference)	(reference)
Female	49,378 (47.52%)	1250 (46.85%)	2837 (46.13%)	HR = 1.14 (95% CI = 0.99–1.32), *p* = 0.077	HR = 0.98 (95% CI = 0.83–1.15), *p* = 0.794	HR = 0.92 (95% CI = 0.84–1.01), *p* = 0.080	HR = 0.91 (95% CI = 0.82–1.01), *p* = 0.068
*Race*							
White	83,823 (80.67%)	2137 (80.10%)	4936 (80.26%)	(reference)	(reference)	(reference)	(reference)
Black	12,638 (12.16%)	355 (13.31%)	793 (12.89%)	HR = 1.09 (95% CI = 0.89–1.35), *p* = 0.397	HR = 0.98 (95% CI = 0.76–1.26), *p* = 0.862	HR = 1.36 (95% CI = 1.20–1.54), *p* < 0.001*	HR = 1.30 (95% CI = 1.12–1.51), *p* = 0.001*
American Indian/Alaska Native	514 (0.49%)	22 (0.82%)	54 (0.88%)	HR = 1.71 (95% CI = 0.92–3.20), *p* = 0.093	HR = 2.32 (95% CI = 1.19–4.55), *p* = 0.014*	HR = 0.92 (95% CI = 0.57–1.51), *p* = 0.756	HR = 0.79 (95% CI = 0.44–1.41), *p* = 0.426
Asian	4294 (4.13%)	74 (2.77%)	204 (3.32%)	HR = 0.77 (95% CI = 0.47–1.27), *p* = 0.304	HR = 0.67 (95% CI = 0.38–1.17), *p* = 0.157	HR = 0.87 (95% CI = 0.65–1.15), *p* = 0.320	HR = 0.85 (95% CI = 0.60–1.19), *p* = 0.343
Pacific Islander	81 (0.08%)	2 (0.07%)	12 (0.20%)	HR = 1.79 (95% CI = 0.25–12.72), *p* = 0.562	HR = 5.89 (95% CI = 0.79–44.00), *p* = 0.084	HR = 0.75 (95% CI = 0.24–2.32), *p* = 0.616	HR = 0.78 (95% CI = 0.19–3.13), *p* = 0.726
Other	1627 (1.57%)	56 (2.10%)	101 (1.64%)	HR = 0.64 (95% CI = 0.35–1.17), *p* = 0.146	HR = 0.87 (95% CI = 0.46–1.61), *p* = 0.650	HR = 0.76 (95% CI = 0.51–1.14), *p* = 0.192	HR = 0.98 (95% CI = 0.63–1.53), *p* = 0.938
*Ethnicity*							
Non‐Hispanic	93,946 (90.41%)	2331 (87.37%)	5475 (89.02%)	(reference)	(reference)	(reference)	(reference)
Hispanic	8464 (8.15%)	314 (11.77%)	611 (9.93%)	HR = 0.59 (95% CI = 0.45–0.77), *p* < 0.001*	HR = 0.67 (95% CI = 0.48–0.94), *p* = 0.020*	HR = 0.76 (95% CI = 0.65–0.90), *p* = 0.002*	HR = 0.86 (95% CI = 0.70–1.05), *p* = 0.146
*Insurance status*							
Private insurance	2843 (2.74%)	97 (3.64%)	206 (3.35%)	(reference)	(reference)	(reference)	(reference)
Not insured	36,971 (35.58%)	934 (35.01%)	2371 (38.55%)	HR = 1.51 (95% CI = 0.95–2.38), *p* = 0.080	HR = 1.62 (95% CI = 1.00–2.63), *p* = 0.050*	HR = 1.69 (95% CI = 1.30–2.19), *p* < 0.001*	HR = 1.57 (95% CI = 1.16–2.12), *p* = 0.003*
Medicaid	8970 (8.63%)	280 (10.49%)	686 (11.15%)	HR = 1.62 (95% CI = 1.21–2.17), *p* = 0.001*	HR = 1.44 (95% CI = 1.02–2.02), *p* = 0.036*	HR = 1.84 (95% CI = 1.57–2.16), *p* < 0.001*	HR = 1.75 (95% CI = 1.46–2.10), *p* < 0.001*
Medicare	52,871 (50.88%)	1285 (48.16%)	2744 (44.62%)	HR = 2.95 (95% CI = 2.45–3.55), *p* < 0.001*	HR = 1.41 (95% CI = 1.08–1.84), *p* = 0.012*	HR = 2.43 (95% CI = 2.18–2.71), *p* < 0.001*	HR = 1.29 (95% CI = 1.11–1.51), *p* = 0.001*
Other Government insurance	1459 (1.40%)	49 (1.84%)	83 (1.35%)	HR = 1.39 (95% CI = 0.73–2.64), *p* = 0.314	HR = 0.85 (95% CI = 0.41–1.76), *p* = 0.663	HR = 1.75 (95% CI = 1.18–2.60), *p* = 0.005*	HR = 1.39 (95% CI = 0.89–2.17), *p* = 0.151
*Setting*							
Metropolitan	84,725 (84.06%)	2111 (81.10%)	4935 (82.57%)	(reference)	(reference)	(reference)	(reference)
Urban	13,912 (13.80%)	419 (16.10%)	923 (15.44%)	HR = 0.99 (95% CI = 0.81–1.21), *p* = 0.943	HR = 0.84 (95% CI = 0.66–1.07), *p* = 0.151	HR = 0.93 (95% CI = 0.82–1.05), *p* = 0.251	HR = 0.86 (95% CI = 0.74–0.99), *p* = 0.044*
Rural	2151 (2.13%)	73 (2.80%)	119 (1.99%)	HR = 1.07 (95% CI = 0.69–1.65), *p* = 0.764	HR = 0.79 (95% CI = 0.48–1.32), *p* = 0.371	HR = 1.05 (95% CI = 0.76–1.43), *p* = 0.778	HR = 0.98 (95% CI = 0.68–1.40), *p* = 0.894
*Charlson‐Deyo score*							
0	70,774 (68.11%)	1620 (60.72%)	4010 (65.20%)	(reference)	(reference)	(reference)	(reference)
1	18,136 (17.45%)	539 (20.20%)	1093 (17.77%)	HR = 1.37 (95% CI = 1.13–1.66), *p* = 0.001*	HR = 1.15 (95% CI = 0.94–1.42), *p* = 0.185	HR = 1.18 (95% CI = 1.05–1.34), *p* = 0.007*	HR = 1.01 (95% CI = 0.88–1.16), *p* = 0.837
2	7278 (7.00%)	248 (9.30%)	491 (7.98%)	HR = 1.75 (95% CI = 1.38–2.21), *p* < 0.001*	HR = 1.25 (95% CI = 0.95–1.63), *p* = 0.105	HR = 1.70 (95% CI = 1.46–1.98), *p* < 0.001*	HR = 1.35 (95% CI = 1.14–1.60), *p* < 0.001*
≥ 3	7725 (7.43%)	261 (9.78%)	556 (9.04%)	HR = 2.42 (95% CI = 1.96–2.98), *p* < 0.001*	HR = 1.91 (95% CI = 1.52–2.40), *p* < 0.001*	HR = 2.06 (95% CI = 1.80–2.35), *p* < 0.001*	HR = 1.45 (95% CI = 1.24–1.69), *p* < 0.001*
*Adults who did not graduate highschool in the area*							
< 5.0%	19,099 (22.00%)	411 (18.25%)	931 (18.32%)	(reference)	(reference)	(reference)	(reference)
5.0%–9.0%	25,467 (29.34%)	673 (29.88%)	1487 (29.26%)	HR = 0.77 (95% CI = 0.61–0.98), *p* = 0.032*	HR = 0.75 (95% CI = 0.58–0.96), *p* = 0.021*	HR = 1.19 (95% CI = 1.02–1.39), *p* = 0.025*	HR = 1.16 (95% CI = 0.99–1.37), *p* = 0.067
9.1%–15.2%	24,131 (27.80%)	643 (28.55%)	1499 (29.50%)	HR = 0.93 (95% CI = 0.74–1.17), *p* = 0.535	HR = 0.82 (95% CI = 0.63–1.07), *p* = 0.147	HR = 1.18 (95% CI = 1.02–1.38), *p* = 0.031*	HR = 1.16 (95% CI = 0.97–1.38), *p* = 0.102
≥ 15.3%	18,115 (20.87%)	525 (23.31%)	1165 (22.92%)	HR = 0.99 (95% CI = 0.78–1.26), *p* = 0.922	HR = 0.92 (95% CI = 0.67–1.25), *p* = 0.587	HR = 1.33 (95% CI = 1.14–1.56), *p* < 0.001*	HR = 1.27 (95% CI = 1.04–1.55), *p* = 0.019*
*Median household income*							
> $74,063	32,974 (38.08%)	722 (32.16%)	1636 (32.24%)	(reference)	(reference)	(reference)	(reference)
$57,857–$74,062	21,311 (24.61%)	588 (26.19%)	1325 (26.11%)	HR = 1.12 (95% CI = 0.91–1.39), *p* = 0.286	HR = 1.11 (95% CI = 0.88–1.39), *p* = 0.384	HR = 0.97 (95% CI = 0.85–1.11), *p* = 0.639	HR = 0.86 (95% CI = 0.74–0.99), *p* = 0.038*
$46,277–$57,856	18,173 (20.98%)	511 (22.76%)	1220 (24.04%)	HR = 1.09 (95% CI = 0.87–1.37), *p* = 0.443	HR = 1.10 (95% CI = 0.84–1.43), *p* = 0.496	HR = 0.99 (95% CI = 0.87–1.13), *p* = 0.891	HR = 0.87 (95% CI = 0.75–1.02), *p* = 0.094
< $46,277	14,144 (16.33%)	424 (18.89%)	893 (17.60%)	HR = 1.47 (95% CI = 1.17–1.83), *p* = 0.001*	HR = 1.39 (95% CI = 1.03–1.87), *p* = 0.031*	HR = 1.30 (95% CI = 1.13–1.49), *p* < 0.001*	HR = 1.02 (95% CI = 0.85–1.23), *p* = 0.831

Abbreviations: CI, confidence interval; HR, hazard ratio; OR, odds ratio.

* Statistically significant result.

**FIGURE 1:**
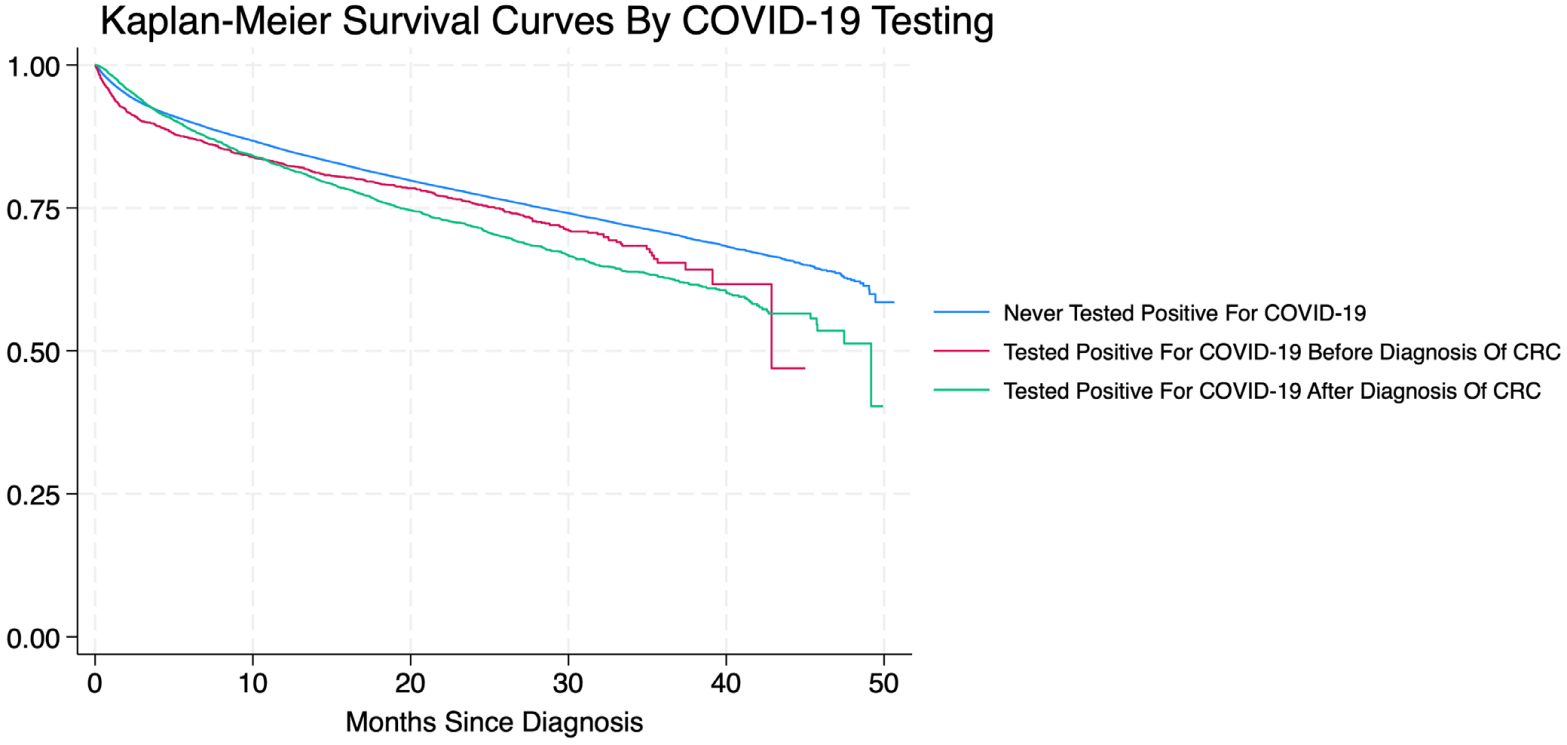
Kaplan–Meier survival curves of patients with CRC (2020–2021). Log‐rank test: *p* < 0.001.

Among those who had COVID‐19 before diagnosis, there was no significant difference in mortality by sex or by setting compared to a metropolitan setting. American Indians with COVID‐19 before diagnosis had higher mortality than their white counterparts (HR = 2.32, 95% CI = 1.19–4.55, *p* = 0.014). Hispanic patients who had COVID‐19 before diagnosis had reduced mortality (HR = 0.67, 95% CI = 0.48–0.94, *p* = 0.020) compared to non‐Hispanics. There was also increased mortality among uninsured (HR = 1.62, 95% CI = 1.00–2.63, *p* = 0.050), Medicaid (HR = 1.44, 95% CI = 1.02–2.02, *p* = 0.036), and Medicare patients (HR = 1.41, 95% CI = 1.08–1.84, *p* = 0.012) compared to patients with private insurance. Patients with a Charlson‐Deyo comorbidity index of ≥ 3 had significantly increased mortality (HR = 1.91, 95% CI = 1.52–2.40, *p* < 0.001). Areas with 5.0%–9.0% high school non‐graduates had lower mortality (HR = 0.75, 95% CI = 0.58–0.96, *p* = 0.021) than areas with < 5.0% non‐graduates. Furthermore, patients with a median household income < $46,277 had higher mortality (HR = 1.39, 95% CI = 1.03–1.87, *p* = 0.031) than those for which it was > $74,063. The log‐rank test for Kaplan–Meier methods and their comparisons showed a *p*‐value of 0.001, demonstrating a significant difference in overall survival among our three cohorts.

We also assessed predictors of higher mortality in those who tested positive for COVID‐19 after being diagnosed with CRC. Like those with COVID‐19 before diagnosis, there was no significant difference in mortality between sexes. For race, only black patients had higher mortality when compared to whites (HR = 1.30, 95% CI = 1.12–1.51, *p* < 0.001). Unlike our other group, there was also no difference in mortality in Hispanics compared to non‐Hispanics (HR = 0.86, 95% CI = 0.70–1.05, *p* = 0.146). However, similar to our other group, there was increased mortality among uninsured (HR = 1.57, 95% CI = 1.16–2.12, *p* = 0.003), those with Medicaid (HR = 1.75, 95% CI = 1.46–2.10, *p* < 0.001), and Medicare patients (HR = 1.29, 95% CI = 1.11–1.51, *p* = 0.001). Residing in an urban setting was associated with lower mortality (HR = 0.86, 95% CI = 0.74–0.99, *p* = 0.044) than residing in metropolitan areas. There was a significant increase in mortality for Charlson‐Deyo comorbidity index of 2 (HR = 1.35, 95% CI = 1.14–1.60, *p* < 0.001) and ≥ 3 (HR = 1.45, 95% CI = 1.24–1.69, *p* < 0.001). Unlike those with COVID‐19 before CRC diagnosis, this group had increased mortality in patients from areas with ≥ 15.3% non‐graduates (HR = 1.27, 95% CI = 1.04–1.55, *p* = 0.019). We also observed decreased mortality in patients in the median household income bracket of $57,857–$74,062 (HR = 0.86, 95% CI = 0.74–0.99, *p* = 0.038).

Our findings suggest that CRC patients with COVID‐19 have worse outcomes than those who have never tested positive for it. Even though both patients with COVID‐19 before diagnosis and after diagnosis of CRC had higher overall mortality, when stratified by stage, only those with COVID‐19 after diagnosis of stage 1, 2, or 3 CRC had a higher mortality. There was no significant difference in mortality for stage 4 disease, likely because these patients had a poor prognosis independent of the COVID‐19 infection. Those who tested positive with COVID‐19 after CRC also experienced treatment delays consistent with what is known about the effect of COVID‐19 infection on cancer care [10]. Violante et al. reported that patients in the “post‐COVID” era presented with advanced‐stage CRC and noted significant staging inequities [11]. We noted in our study that race, ethnicity, setting, education status, and income had a variable association with mortality depending on when patients tested positive. Interestingly, Hispanic patients with COVID‐19 before CRC had decreased mortality even though they had decreased CRC screening rates during this time [12]. This can be explained by selection bias or lack of power, but may need further exploration. What was consistent between both groups was that patients who were uninsured, had Medicare, or had Medicaid had higher mortality than their counterparts with private insurance. This aligns with a study looking at gynecological cancer by Lara et al. [13]. Another consistent finding was that patients with a higher comorbidity burden had higher mortality. Patients with a higher comorbidity burden have higher mortality anyway, but this may also be due to COVID‐19 exacerbating chronic conditions in addition to possibly its intrinsic effects on CRC [14].

Our study is unique in that it looks at outcomes and predictors for COVID‐19 exclusively in the “post‐COVID” era and only includes patients who were tested and had known results. Our study was limited in that it looked over a relatively short timeframe, 2 years. There is also a possibility of misclassification bias, as patients may have tested positive previously and later tested negative at the facility reporting the case, as well as immortal time bias in patients who died shortly after diagnosis. Furthermore, accessibility to healthcare could be dependent on the wave of COVID‐19, which may be a potential confounder that cannot be quantified with our dataset. It is unclear whether COVID‐19 is a causal agent for this worse prognosis or if COVID‐19 infection indicates poor health outcomes, as patients who get this infection tend to have poorer outcomes anyway [15]. These findings imply that vaccinations may be part of the recommended treatment guidelines. Further, patients with CRC who develop COVID‐19 infection may warrant closer follow‐up during treatment. Future research needs to explore the interaction of COVID‐19 infection and treatment efficacy and toxicity, and other factors on the causal pathway to poor outcomes.

## Author Contributions

**Imran Qureshi:** conceptualization, investigation, writing – original draft, methodology, visualization, writing – review and editing, software, formal analysis, data curation. **Steven Rella:** writing – original draft, writing – review and editing, visualization. **Aasma Shaukat:** supervision, writing – review and editing, methodology, writing – original draft, conceptualization.

## Funding

The authors have nothing to report.

## Ethics Statement

Institutional review board was exempted for this study as it does not directly involve humans.

## Conflicts of Interest

The authors declare no conflicts of interest.

## Data Availability

All data was derived from the National Cancer Database, which is available through an application process to investigators associated with the Commission on Cancer–accredited cancer programs by the American College of Surgeons.
